# Perceived Discrimination Is a Mediator of Rural Identity and Cardiometabolic Multimorbidity Among U.S. Adults

**DOI:** 10.3390/ijerph22030426

**Published:** 2025-03-14

**Authors:** LaToya J. O’Neal, Lisa Scarton, Ara Jo, Biswadeep Dhar, Folakemi T. Odedina, Diana J. Wilkie

**Affiliations:** 1 Department of Family, Youth and Community Sciences, Institute of Food and Agricultural Sciences, College of Agricultural and Life Sciences, University of Florida, Gainesville, FL 32611, USA; bdhar@umes.edu; 2 Department of Family, Community and Health Systems Science, College of Nursing, University of Florida, Gainesville, FL 32611, USA; lscarton@ufl.edu; 3 Department of Health Services Research, Management and Policy, College of Public Health and Health Professions, University of Florida, Gainesville, FL 32611, USA; ara13j@phhp.ufl.edu; 4 Department of Human Ecology, University of Maryland Eastern Shore, Princess Anne, MD 21853, USA; 5 Division of Hematology/Oncology, Mayo Clinic, Jacksonville, FL 32224, USA; odedina.folakemi@mayo.edu; 6 Department of Biobehavioral Nursing Science, College of Nursing, University of Florida, Gainesville, FL 32611, USA; diwilkie@ufl.edu

**Keywords:** rural health, multimorbidity, chronic conditions, cardiometabolic conditions, health disparities, health equity, population health, social determinants of health

## Abstract

The rise in prevalence of cardiometabolic multimorbidity indicates the need for more research examining associated risk factors. Identifying multilevel risk factors is especially critical for U.S. health disparity populations who have been shown to experience a disproportionate burden of chronic disease-related morbidity and mortality. This study examines differences in the prevalence of and risk factors associated with cardiometabolic multimorbidity status among health disparity populations in a representative sample of U.S. adults. Additionally, we investigate the role of perceived discrimination as a mediator of the relationship between rural identity and cardiometabolic multimorbidity status. We report the overall and stratified prevalence of cardiometabolic multimorbidity. Findings from multivariate logistic regression indicated that age, rural identity, healthcare access, and perceived discrimination were associated with higher odds of cardiometabolic multimorbidity. Perceived discrimination was found to be a significant mediator for the relationship between rural identity and cardiometabolic multimorbidity status. These findings have implications for the design and implementation of effective multilevel interventions to reduce the impact of perceived discrimination on cardiometabolic multimorbidity among rural adults.

## 1. Introduction

Multimorbidity is a global public health issue as individuals living with multimorbidity have been found to experience higher health care costs, poor quality of life, greater functional decline, increased risk of mortality, and lower life expectancy [1,2,3,4,5]. The worldwide prevalence of multimorbidity is 37.2% and is highest in South America (45.7%), followed by North America (43.1%), Europe (39.2%), and Asia (35%) [6]. In the United States (U.S.), the prevalence of multimorbidity increased from 21.8% [7] to 27.2% [8] between 2001 and 2018. Cardiometabolic multimorbidity (CM) is an increasing concern as cardiometabolic conditions, such as diabetes, hypertension, and heart disease, are associated with modifiable lifestyle behaviors and are the most common noncommunicable diseases in the world [9].

The prevalence of CM among U.S. adults increased from 9.4% to 14.4% between 2008 and 2018 [10]. With the prevalence of CM continuing to rise, there is a need for additional research to move beyond reporting prevalence and identify the complete range of risk factors associated with CM [11]. This is especially true for minoritized and populations with limited access to resources in the U.S. who often experience a disproportionate burden of health disparities due to higher prevalence of chronic conditions [12,13,14] and CM [10].

Health disparity populations, including rural, low socioeconomic status, and racial/ethnic minorities, often have a higher prevalence of cardiometabolic conditions [10,15]. Given that cardiometabolic risk factors such as dyslipidemia, dysglycemia, obesity, and hypertension are more prevalent among these groups, it is important to describe the prevalence of CM among these populations. Additionally, while international research has identified cardiometabolic risk factors such as having hypertension, overweight, and obesity [16,17]; behavioral and emotional risk factors such as fruit and vegetable consumption, physical activity levels, and stress [18]; biological risk factors such as age and sex [6,19,20]; and geographic factors such as rurality [21] as being associated with higher odds or prevalence of CM, research examining these associations among U.S. adults is limited. Moreover, research examining how the social determinants of health (SDOH), which include multilevel risk factors at the individual, environmental, and societal levels, are associated with CM status and may contribute to differences in CM is also limited.

Previous research has found relationships between socioeconomic status (SES), health behaviors, perceived health status, health care access, discrimination, and prevalence of chronic cardiometabolic conditions [22,23,24,25]. However, there is a gap in knowledge as it relates to the role of SDOH on CM among health disparity populations as well as which SDOH may explain the relationship between health disparity status and CM. The current study aims to examine differences in the prevalence of and risk factors associated with CM among health disparity populations. We examined the role of individual level factors (such as socioeconomic status, health literacy, perceived health status, health behaviors, and rural identity), environmental factors (such as access to health care), and societal level factors (such as perceived discrimination) on CM among a representative sample of U.S. adults. Additionally, we evaluate whether perceived discrimination mediates the association between rural identity and CM among U.S. adults.

## 2. Materials and Methods

### 2.1. Sample

Using a cross-sectional study design, we surveyed 2180 adults through a national panel (*n* = 1750), a state panel (*n* = 330), and a community-engaged sample (*n* = 100). The national sample consisted of panelists who live in the U.S., were aged ≥18, and were selected by Qualitics (who also managed the data collection from the survey). The sample quotas represent the general population in the U.S. The Bureau of Economic and Business Research (BEBR) managed the state sample (*n* = 330). BEBR purchased a panel from a provider who used inclusion criteria that was narrower than the national sample. While participants still needed to be aged ≥ 18, they needed to live within the catchment area of the University of Florida (UF) Health Cancer Center. The community engaged sample (*n* = 100) had the same inclusion criteria as BEBR, but they were recruited through email distribution of flyers as well as Facebook ads. Both samples matched state census data. Participants completed informed consent prior to engaging in the study and were compensated for their participation.

### 2.2. Study Measures

#### 2.2.1. Cardiometabolic Multimorbidity (CM) Status

CM status is a categorical variable indicated by participants responses to whether they had ever been told by a health care provider they had diabetes, heart problems, and high blood pressure. If they responded in the affirmative to two or more conditions, they were coded “yes” for having CM.

#### 2.2.2. Other Measures

Rural identity was measured using the 5-item Rural Identity Scale [26]. It is a five-item measure assessing the sense of belonging and group attitudes. The Likert scale responses ranged from 0 = “not at all” to 6 = “extremely” with total scores ranging from 0 to 30. Higher scores indicate stronger rural identify (sense of belonging to a rural community).

Perceived discrimination was measured using the Everyday Discrimination Scale (Short Version) [27]. Participants were asked about how frequently they experienced discrimination in their everyday lives. The Likert scale responses ranged from 1 = “never” to 6 = “almost every day” (range 1–30) with higher scores indicating higher perceived discrimination.

Additional details of the study design, procedures, and survey instruments have been reported previously [28]. Variables included healthcare access, information seeking and health information access, health status, and health behaviors.

#### 2.2.3. Study Covariates

Covariates such as self-reported demographic data and socioeconomic status were also included in the analysis. Age was a continuous variable. Race was a categorical variable, including Black/African American, White, and other. Hispanic/Latino(a) ethnicity was coded “yes” for Hispanic/Latino(a) and “no” for non-Hispanic/Latino(a). Gender was a categorical variable, male and female. Marital status categories included married and not married. Employment status included the categories of having a disability, working full-time, working part-time, homemaker, retired, student, and unemployed. Education was a categorical variable, including less than high school graduate, high school graduate, some college/technical degree, college graduate, and postgraduate. Income was combined into four categories, $0–$19,999, $20,000–$49,999, $50,000–$99,999, and $100,000 and up.

### 2.3. Ethics Information

This study was approved by the Institutional Review Board at the University of Florida. All participants provided informed consent to participate in this study.

### 2.4. Statistical Analysis

We conducted a bivariate analysis using chi-square tests to compare the prevalence of the variables between those with and without CM. Univariable logistic regression was used to examine the relationship between each variable and CM status. Multivariable logistic regression was used to examine the association between social determinants of health and CM status with covariates. Backward selection was used to select the appropriate covariates. Finally, we investigated whether discrimination is a mediator of the association between rural identity and CM by using linear regression and probit regression models. Data were analyzed using R statistical software [29].

## 3. Results

The demographic and descriptive statistics for the study sample are described in Table 1. Most participants were White (73%) and female (54%). Over half (56%) of participants were married, worked full-time (43%), had a college degree or higher (50.9%), with an annual income over $50,000 (52%).

The overall prevalence of CM in the sample was 11.4%. The prevalence stratified by race/ethnicity was 12.1% among White adults, 12% among Hispanic adults, and 11.1% among Black adults. CM prevalence was also stratified by socioeconomic status (education and income). The prevalence of CM was highest among both the highest income group (13.4%) and education level (13.3%) when compared to the lowest income group (11.4%) and the lowest education level (9.1%).

Table 2 indicates the prevalence of CM by health disparity status, social determinants of health, health behaviors, and other covariates (only significant variables are shown). CM prevalence is presented in columns with “no” representing those reporting less than two chronic conditions and “yes” representing those with two or more cardiometabolic conditions (diabetes, heart problems, and high blood pressure). The prevalence of CM varies significantly by employment status, health care access, confidence obtaining health information, and overall health status.

Table 3 shows CM status by continuous variables (only significant variables are shown). Significant differences exist by age and rural identity.

### Multivariate Logistic Regression

Table 4 presents the results of the multivariate logistic regression analysis (only significant variables are shown). In the adjusted multivariate model, older adults had higher odds of having CM (OR:1.04, 95%CI: 1.03–1.05, *p* < 0.001). In addition, those with a stronger rural identity were more likely to have CM (OR: 1.03, 95% CI: 1.01–1.05, *p* = 0.001). Individuals who had an inability to access care reported significantly higher odds of having CM (OR: 1.95, 95% CI: 1.29–2.94, *p* = 0.002). Those experiencing discrimination had higher odds of CM (OR: 1.04, 95% CI: 1.01–1.07, *p* = 0.02). Perceived health status was significantly related to CM. Specifically, compared to those reporting excellent health, the odds of CM increased as reported health status declined. Individuals who reported poor health status had eight times greater odds of CM when compared to those reporting excellent health (OR: 8.80, 95% CI: 3.35–22.68, *p* < 0.001). Overall, confidence obtaining health information was significantly associated with CM.

We also conducted a mediation effect analysis to determine whether discrimination mediated the relationship between rurality and CM status. The total effect between rural identity and CM showed a significant association (OR: 1.03, 95%CI: 1.02–1.05, *p* < 0.001). In the regression model testing the mediation effect, rural identity was significantly associated with discrimination (*p* < 0.001). Additionally, the adjusted probit model displayed a significant direct effect between rural identity and CM after controlling discrimination (*p* = 0.001), and discrimination was significantly associated with CM indicating a significant indirect effect (*p* = 0.018). As a result, discrimination was found to be a significant mediator for the relationships between rural identity and CM status.

## 4. Discussion

### 4.1. Summary of Findings

This study examined differences in the prevalence of and risk factors associated with cardiometabolic multimorbidity (CM) in a representative sample of U.S. adults. We identified the prevalence of CM by race/ethnicity and socioeconomic status and observed differences in the prevalence of and multilevel social determinants of health associated with CM. While CM prevalence did not vary significantly by race, ethnicity, or socioeconomic status, Hispanic ethnicity was associated with higher odds of CM. Older age, less than excellent health status, inability to access healthcare, rural identity, and perceived discrimination were also associated with higher odds of CM. Our most significant finding is that discrimination is a mediator for rural identity and CM.

### 4.2. Comparison with Previous Studies

In this study, the prevalence of CM was 11.4% among a representative sample of U.S. adults. We stratified CM prevalence by race, ethnicity, and socioeconomic status (SES; education and income). Prevalence did not vary significantly between health disparity populations as White and Hispanic adults had a CM prevalence of about 12% and the CM prevalence among Black adults was about 11%. For SES, those at the highest income and education levels had a higher CM prevalence (13.4% and 13.3% respectively) when compared to those at the lowest income and education levels (11.4% and 9.1% respectively). The overall CM prevalence rates in the current study were slightly lower than a recent study, and they found CM prevalence was higher in Non-Hispanic Black adults [10]. Differences in prevalence between the two studies may be the result of differences in the measurement of CM as the current study used two or more (maximum of three) conditions and the other study calculated age-standardized prevalence using three or more cardiometabolic conditions [10]. Still, both studies confirm the high prevalence of CM in the U.S. population as a critical public health concern [30,31].

Additionally, we found individual level factors such as older age, less than excellent health status, and rural identity; environmental level factors such as inability to access healthcare; and societal level factors such as perceived discrimination were associated with higher odds of CM. Several studies confirm that older age is associated with CM [16,32,33]. This finding confirms that as individuals age, they have higher morbidity and establishes the importance of primary and secondary prevention efforts as there is a critical period for intervention to reduce the burden of CM. The association between self-reported health status and CM posits that individuals with CM experience physical health decline. This finding confirms other studies that show those who have more than one chronic condition experience greater challenges to physical functioning and report poorer health status [4,34,35].

Geographic disparities, including rurality, have been found to be associated with CM [21]. This study examines the role of rural identity, a measure of sense of belonging and group attitudes. Rural identity is important for assessing how an individual perceives themselves and the social group to which they belong despite their zip code. Having a stronger connection to rural identity has implications for the development of tailored interventions that consider the role of cultural norms, diversity, and intersectionality to inform public health practice [36]. Finding that rural identity is associated with higher odds of CM is consistent with the literature on the prevalence of cardiometabolic conditions in general within rural populations [37,38]. Our finding confirms the association between rurality and higher odds of CM among a nationally representative sample. Inability to access care is a barrier to overall health. Finding that those who have difficulty accessing healthcare have higher odds of comorbidity confirms the importance of health care access for those with CM, especially among rural populations [39].

The most significant finding in this study is that discrimination is associated with higher odds of CM and is a mediator for rural identity and CM [37,38]. Research has shown that racial discrimination has negative effects on the health of minoritized populations [40]. Similarities among cardiometabolic risk factors between health disparity populations, like poor nutrition, physical inactivity, limited resources, and limited healthcare access [41] may explain feelings of discrimination across these groups. Our findings suggest that perceived discrimination may also be impacting the health of rural populations. Yet, there are no studies, to our knowledge, examining perceived discrimination by geographic residence. Additional research is needed to understand how the health of rural adults is impacted by perceived discrimination.

### 4.3. Strengths and Limitations

This study has notable strengths and limitations. While there has been more research focused on multimorbidity in recent years, the U.S. literature is limited as it relates to social determinants of health (SDOH) and CM among health disparity populations. This study contributes to the literature by identifying multilevel risk factors associated with CM. This study also has translational value as local sampling focused on the UF Health catchment area. Understanding the risk factors associated with CM in the local area will allow for the development of tailored and targeted interventions that will better meet the needs of UF Health patients with CM. As with all cross-sectional analyses, we are unable to establish causal relationships. Likewise, with self-reported data, there is the risk of bias in responses. While we aimed to assess differences by health disparity populations, response distributions limited our ability to analyze differences between groups. In addition, the measures of healthy eating in this study were limited to single item responses, which may have impacted the validity of the measures and contributed to lack of significant findings between healthy eating behaviors and CM. Finally, this sample reported higher than average education and income. This might have influenced study findings as we aimed to examine CM among health disparity populations.

## 5. Conclusions

This study reports the prevalence of CM in a nationally representative sample. CM prevalence did not differ significantly by health disparity status. However, social determinants of health at the individual, environmental, and societal levels were associated with higher odds of CM. Of particular note is that perceived discrimination emerged as a significant mediator for rural identity and CM. Our findings have implications for the design and implementation of effective multilevel interventions to reduce the impact of SDOH, especially discrimination, and improve cardiometabolic health among rural adults.

## Figures and Tables

**Table 1:** Demographics and Descriptive Statistics of Study Population.

Variable	Mean/Percentage
Age (*n* = 2225)	45.8
Race (*n* = 2135)	
White	73
Black	14
Other	9
Ethnicity (*n* = 2202)	
Non-Hispanic, Latino/a	82
Hispanic, Latino/a	17
Gender (*n* = 2220)	
Female	54
Male	45
Marital Status (*n* = 2206)	
Married	56
Not Married	43
Employment Status (*n* = 2193)	
Disabled	4.9
Full-Time	43.2
Part-Time	10.9
Homemaker	4.9
Retired	20.3
Student	4.9
Unemployed	9.2
Education (*n* = 2200)	
<High School Graduate	3.9
High School Graduate	18.2
Some College/Tech	25.8
College Graduate	28.4
Postgraduate	22.5
Income (*n* = 2096)	
$0–$19,999	16.5
$20,000–$49,000	25.3
$50,000–$99,999	28.1
$100,000 or more	24.1

**Table 2:** Cardiometabolic Multimorbidity (CM) Status by Categorical Risk Factors.

Variable	Category	Cardiometabolic Multimorbidity		*p*-Value
		No	Yes	
Employment Status	Disabled	84 (4.50%)	23 (9.40%)	<0.001 *
	Employed Full-Time	839 (44.60%)	94 (38.50%)	
	Employed Part-Time	212 (11.30%)	25 (10.20%)	
	Homemaker	94 (5%)	11 (4.50%)	
	Retired	368 (19.60%)	73 (29.90%)	
	Student	99 (5.30%)	4 (1.60%)	
	Unemployed	186 (9.90%)	14 (5.70%)	
Healthcare Access	No	1290 (70.90%)	146 (60.60%)	0.001 *
	Yes	530 (29.10%)	95 (39.40%)	
Confidence Obtaining Health Information	Not Confident	44 (2.40%)	3 (1.20%)	0.029 *
	A little Confident	100 (5.40%)	6 (2.50%)	
	Somewhat Confident	330 (17.90%)	36 (14.90%)	
	Very Confident	613 (33.20%)	75 (31%)	
	Completely Confident	757 (41.10%)	122 (50.40%)	
Overall Health Status	Excellent	411 (21.60%)	43 (17.50%)	<0.001 *
	Very Good	615 (32.30%)	38 (15.40%)	
	Good	590 (31%)	88 (35.80%)	
	Fair	242 (12.70%)	62 (25.20%)	
	Poor	45 (2.40%)	15 (6.10%)	

* Indicates significance.

**Table 3:** Cardiometabolic Multimorbidity (CM) Status by Continuous Risk Factors.

Variable	Cardiometabolic Multimorbidity		*p*-Value
	No Mean (SD)	Yes Mean (SD)	
Age	45 (17.6)	52.5 (16.5)	<0.001 *
Rural Identity	16 (9.2)	18.4 (8.4)	<0.001 *

* Indicates significance.

**Table 4:** Multivariate Logistic Regression by Cardiometabolic Multimorbidity (CM) Status.

Variable	OR	*p*-Value	95% CI	Overall *p*-Value
Age	1.04	<0.001 *	1.03–1.05	
Hispanic	1.90	0.012 *	1.14–3.10	
Rural Identity	1.03	0.001 *	1.01–1.05	
Inability to Access Healthcare Yes vs. No	1.95	0.002 *	1.29–2.94	
Discrimination	1.04	0.02 *	1.01–1.07	
Overall, Health Status				<0.001 *
Health Status Good vs. Excellent	3.28	<0.001 *	1.9–5.8	
Health Status Fair vs. Excellent	5.54	<0.001 *	3.01–10.4	
Health Status Poor vs. Excellent	8.80	<0.001 *	3.35–22.68	
Overall, Confidence Obtaining Health Information (COHI)				0.008 *
COHI A little vs. not at all	0.80	0.12–6.68	0.815	
COHI Somewhat vs. not at all	2.22	0.56–15.04	0.32	
COHI Very vs. not at all	3.08	0.8–20.5	0.153	
COHI Completely vs. not at all	3.88	1.01–25.75	0.085	

* Indicates significance.

## Data Availability

The raw data supporting the conclusions of this article will be made available by the authors on request.

## Abbreviations

The following abbreviations are used in this manuscript:

MDPI	Multidisciplinary Digital Publishing Institute
DOAJ	Directory of open access journals
TLA	Three-letter acronym
LD	Linear dichroism
